# ICRAC controls the rapid androgen response in human primary prostate epithelial cells and is altered in prostate cancer

**DOI:** 10.18632/oncotarget.1483

**Published:** 2013-10-28

**Authors:** Christian Holzmann, Tatiana Kilch, Sven Kappel, Andrea Armbrüster, Volker Jung, Michael Stöckle, Ivan Bogeski, Eva C. Schwarz, Christine Peinelt

**Affiliations:** ^1^ Department of Biophysics, Saarland University, Homburg, Germany; ^2^ Clinics of Urology and Pediatric Urology, Saarland University, Homburg, Germany

**Keywords:** Membrane androgen receptor, Orai channel, CRAC channel, prostate cancer, Ca^2+^ signaling

## Abstract

Labelled 5α-dihydrotestosterone (DHT) binding experiments have shown that expression levels of (yet unidentified) membrane androgen receptors (mAR) are elevated in prostate cancer and correlate with a negative prognosis. However, activation of these receptors which mediate a rapid androgen response can counteract several cancer hallmark functions such as unlimited proliferation, enhanced migration, adhesion and invasion and the inability to induce apoptosis. Here, we investigate the downstream signaling pathways of mAR and identify rapid DHT induced activation of store-operated Ca^2+^ entry (SOCE) in primary cultures of human prostate epithelial cells (hPEC) from non-tumorous tissue. Consequently, down-regulation of Orai1, the main molecular component of Ca^2+^ release-activated Ca^2+^ (CRAC) channels results in an almost complete loss of DHT induced SOCE. We demonstrate that this DHT induced Ca^2+^ influx via Orai1 is important for rapid androgen triggered prostate specific antigen (PSA) release. We furthermore identified alterations of the molecular components of CRAC channels in prostate cancer. Three lines of evidence indicate that prostate cancer cells down-regulate expression of the Orai1 homolog Orai3: First, Orai3 mRNA expression levels are significantly reduced in tumorous tissue when compared to non-tumorous tissue from prostate cancer patients. Second, mRNA expression levels of Orai3 are decreased in prostate cancer cell lines LNCaP and DU145 when compared to hPEC from healthy tissue. Third, the pharmacological profile of CRAC channels in prostate cancer cell lines and hPEC differ and siRNA based knock-down experiments indicate changed Orai3 levels are underlying the altered pharmacological profile. The cancer-specific composition and pharmacology of CRAC channels identifies CRAC channels as putative targets in prostate cancer therapy.

## INTRODUCTION

In classical steroid receptor pathways, hormones cross the plasma membrane and bind to their cytosolic receptors. Subsequently, these complexes translocate to the nucleus where they trigger gene expression important for many physiological and pathophysiological functions and thus are targets for therapeutic strategies [1-3]. In contrast to the classical pathway where the hormonal effects appear after hours, many cell types display rapid hormone signaling upon steroid hormone stimulation mediated by receptors or ion channels located at the cell surface [4, 5].

Even though the molecular identity of mAR is still elusive, their presence has been demonstrated in the membrane of primary prostate tissue and was correlated to the level of differentiation of prostate carcinoma [6, 7]. mAR are existent in androgen-sensitive prostate cancer cell line LNCaP [8] as well as in androgen-insensitive prostate cancer cell lines DU145 and PC3 [9, 10]. Rapid DHT signaling results in rearrangements of the cytoskeleton, PSA production, inhibition of proliferation, migration, adhesion and invasion and apoptotic regression of prostate cancer cells [8, 11, 12]. Several studies in mice demonstrated the clinical relevance of targeting mAR in prostate cancer therapy. Macroscopic tumors are reduced upon treatment with testosterone-albumin conjugates, binding exclusively to mAR. In addition, testosterone-BSA triggers tumor cell apoptosis as the fraction of apoptotic cells in tumorous tissue is elevated. Co-medication of mice with paclitaxel and testosterone-BSA results in additive tumor inhibitory rates up to ~92% [11, 12]. Taken together, targeting mAR pathways in prostate cancer is a highly promising strategy especially as no toxic effects of testosterone-albumin conjugates have been reported in these studies [13].

As a universal mechanism rapid androgen signaling includes an increase in intracellular Ca^2+^ as second messenger [4]. Previous work proposed that the mAR induced increase in intracellular Ca^2+^ arises from intracellular Ca^2+^ store depletion and the Ca^2+^ influx via voltage gated Ca^2+^ channels in the plasma membrane in LNCaP cells [14].

During the last few years the molecular components of SOCE and the underlying Ca^2+^ current I_CRAC_ (Ca^2+^ release-activated Ca^2+^ current) have been identified: stromal interaction molecule STIM1 [15, 16] and plasma membrane protein Orai1 [17-19]. Upon Ca^2+^ release from intracellular Ca^2+^ stores, Ca^2+^ dissociates from an EF hand motif in the luminal section of STIM1. STIM1 molecules cluster and activate Ca^2+^ influx via Orai1 ion channels in the plasma membrane [20-24]. A number of studies of the STIM1 homologue STIM2 and the Orai1 homologues Orai2 and Orai3 increasingly reveal disease related roles for these less prominent but ubiquitously expressed isoforms [25].

I_CRAC_ mediates a plethora of cellular functions including cell cycle regulation, proliferation and apoptosis [26]. In prostate cancer, Ca^2+^ signaling via I_CRAC_ channels is decreased and subsequently, the low I_CRAC_ contributes to cancer hallmark functions in particular uninhibited proliferation and the inability to induce apoptosis [27-29]. In addition, low expression levels of Orai1 can protect LNCaP cells from several apoptotic pathways [30].

Here, we investigate the role of I_CRAC_ channel components in Ca^2+^ signaling in the rapid response to DHT stimulation. We compare expression levels of STIM1, STIM2, Orai1, Orai2 and Orai3 in tumorous and non-tumorous tissue from prostate cancer patients. In addition, we examine the pharmacological profile of I_CRAC_ in hPEC from non-tumorous tissue and prostate cancer cell lines LNCaP and DU145 to investigate I_CRAC_'s molecular key players as potential therapeutic targets.

## RESULTS

### DHT induces SOCE in hPEC

**Figure 1:**
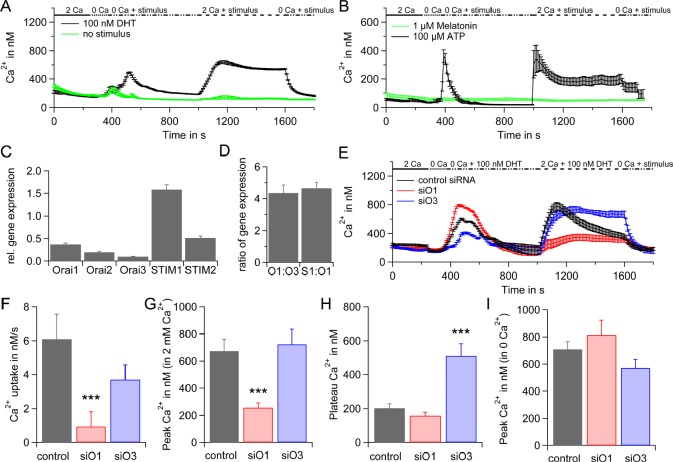
SOCE in hPEC A) Average intracellular Ca^2+^ responses (±SEM) from a Fura-2 based Ca^2+^ imaging assay when 100 nM DHT, (n = 44) or no stimulus (n = 14) were applied are blotted vs. time. Extracellular Ca^2+^ concentration is indicated in mM. B) Same as A but either 100 μM ATP, n = 37 (black), or 1 μM Melatonin, n = 14 (green) were used as stimulus. C) qRT-PCR analyses of Orai1, Orai2, Orai3, STIM1 and STIM2 expression levels from hPEC from 17 different patients normalized to TATA box binding protein (TBP) expression as reference gene. D) Ratio of Orai1:Orai3 and STIM1:Orai1 expression levels. E) DHT induced intracellular Ca^2+^ responses in cells transfected with control RNA (black, n = 38), Orai1 siRNA (red, n = 33) or Orai3 siRNA (blue, n = 36). F) Average Ca^2+^ influx rates for cells in E when 2 mM Ca^2+^ and 100 nM DHT were applied. G) Average of Ca^2+^ peaks for cells in E when 2 mM Ca^2+^ and 100 nM DHT were applied when baseline for every cell was subtracted. H) Average Ca^2+^ plateaus for cells in E when 2 mM Ca^2+^ and 100 nM DHT were applied at t = 1600 s and baseline for every cell was subtracted. I) Average Ca^2+^ peaks after store depletion with 100 nM DHT in Ca^2+^ free Ringer for cells in E when baseline for every cell was subtracted.

First, we investigate the molecular key players in androgen induced Ca^2+^ signaling in hPEC. Application of 100 nM DHT in Ca^2+^ free solution induces a substantial increase in intracellular Ca^2+^ due to Ca^2+^ release from intracellular Ca^2+^ stores as has been described earlier [14]. The subsequent addition of 2 mM Ca^2+^ induced a rapid increase in intracellular Ca^2+^ concentration thus confirming that DHT induces SOCE in hPEC. Control cells on which no DHT has been applied release Ca^2+^ from intracellular Ca^2+^ stores to some extent, possibly induced by the Ca^2+^ free solution. But both, Ca^2+^ release from intracellular Ca^2+^ stores and SOCE are almost reduced to zero in control cells (Figure 1A). Supplementary Figure 1A shows that store-depletion by sarco-/endoplasmic reticulum Ca^2+^-ATPase (SERCA) inhibitor thapsigargin (tg) evokes SOCE, confirming the principle mechanism of SOCE in hPEC.

Adenosine-5'-triphosphate (ATP) has been described as signal molecule for prostate epithelial cells [31] as well as melatonin [32]. Application of 100 μM ATP activates SOCE but 1 μM melatonin does not (Figure 1B) suggesting that ATP induced signaling includes SOCE pathways whereas melatonin signaling pathways do not.

Please note that basal Ca^2+^ levels vary between 100 nM and 200 nM (Figure 1A and 1B and Supplementary Figure 1A) most likely as data were generated with cell preparations from different patients.

Based on these initial findings we analysed gene expression levels of CRAC channel components Orai1, Orai2, Orai3, STIM1 and STIM2 by qRT-PCR in hPEC from 17 different patients (Figure 1C and Supplementary 1B). Our data suggest that CRAC channels in hPEC are mainly formed by STIM1 and Orai1. Figure 1D represents the ratio of Orai1:Orai3 and STIM1:Orai1 expression pointing towards an STIM1:Orai1 ratio of 4.6±0.4, which, assuming a linear correlation between mRNA and protein levels would be above optimal for maximal SOCE activation [33] and a relatively low Orai1:Orai3 ratio of 4.3±0.5 (for comparison, the Orai1:Orai3 ratio is ~70 in naïve and ~25 in effector T_H_ cells, [34]). The latter points towards a contribution of approximately one Orai3 subunit to the functional CRAC channel, that has been described as either tetramer [35-40] or hexamer [41] in the past.

The down-regulation of the SOCE component Orai1 by siRNA significantly reduced the Ca^2+^ influx rate and peak of SOCE and the Ca^2+^ plateau is decreased when compared to SOCE in cells transfected with non-silencing control RNA (Figure 1E, F, G and H). The down-regulation of Orai3 had little effect on the Ca^2+^ influx rate and peak of SOCE, but significantly increased the Ca^2+^ plateau (Figure 1E, F, G and H). Knock-down efficiencies are shown in Fig. S1c. In order to investigate if down-regulation of Orai1 or Orai3 leads to differences in Ca^2+^ release from intracellular Ca^2+^ stores, we subtracted base lines from DHT induced Ca^2+^ peaks in 0 Ca^2+^ for single cells and analysed the averages. The differences in the degree of store depletion are not significant (Figure 1I).

In conclusion, these data show that rapid DHT response involves Ca^2+^ signaling via I_CRAC_ channels and indicate a key role for Orai1 whereas the function of Orai3 is less clear.

### Molecular components of ICRAC mediate rapid DHT response

**Figure 2:**
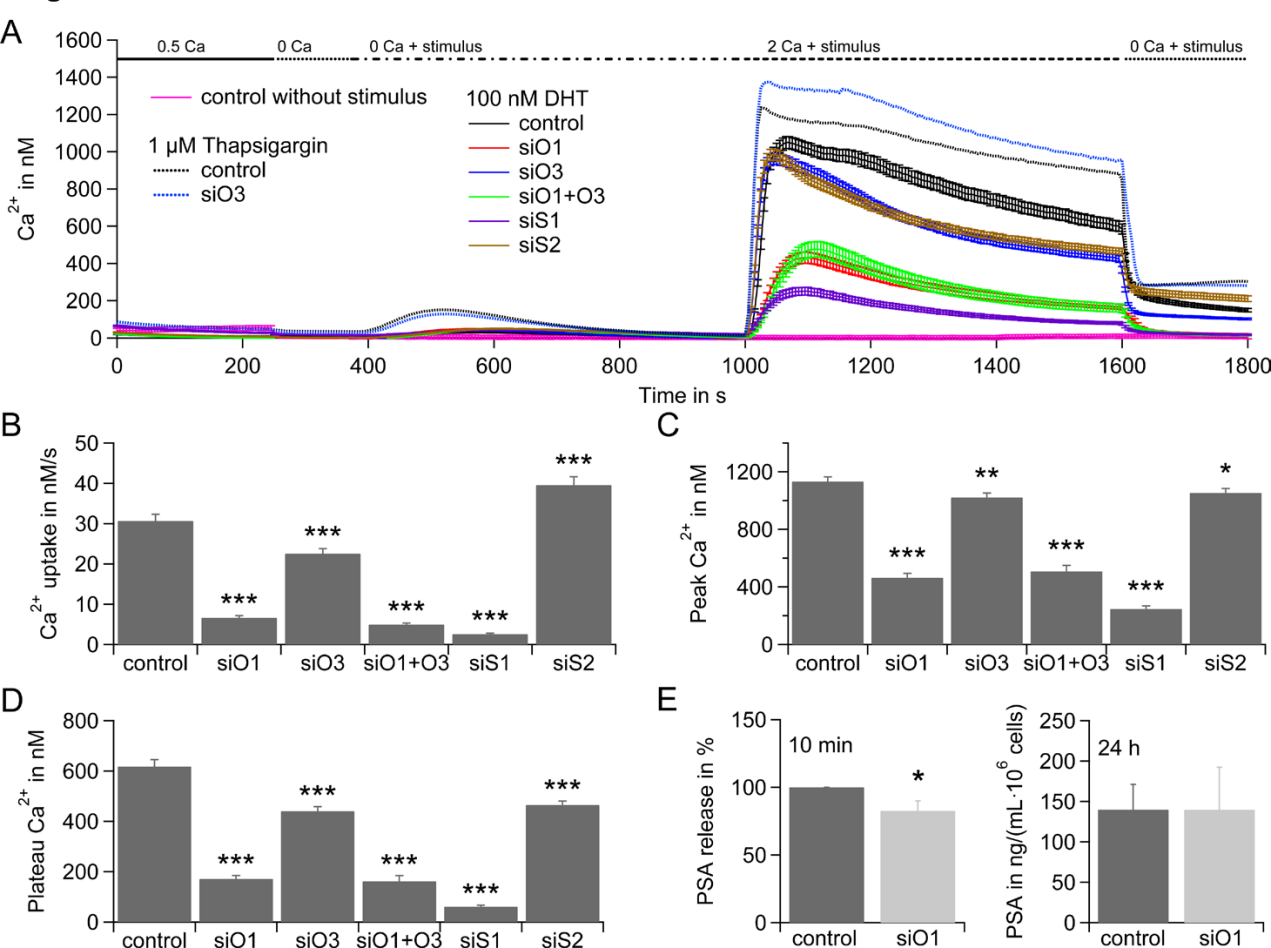
Rapid DHT response in LNCaP depends on SOCE A) Average intracellular Ca^2+^ responses (±SEM) from a Fura-2 based Ca^2+^ imaging assay when stores are depleted by application of 100 nM DHT (solid lines) and cells were transfected with control RNA (control, black curve, n = 132), Orai1 siRNA (siO1, red curve, n = 117), Orai3 siRNA (siO3, blue curve, n = 126), Orai1 and Orai3 siRNA (siO1+O3, green curve, n = 77), STIM1 siRNA (siS1, purple curve, n = 137), STIM2 siRNA (siS2, brown curve, n = 180), or stores are depleted by application of 1 μM tg (dotted line) and cells were transfected with control RNA (control, black curve, n = 228) or Orai3 siRNA (siO3, blue curve, n = 227) or stores were not depleted (no stimulus) and transfected with control RNA (pink curve, n = 45). Extracellular Ca^2+^ concentration is indicated in mM. B) Average Ca^2+^ influx rates from cells in A, when 100 nM DHT and 2 mM Ca^2+^ were applied. C) Average Ca^2+^ peaks from cells in A, when 100 nM DHT and 2 mM Ca^2+^ were applied and baseline for every cell was subtracted. D) Average Ca^2+^ plateaus from cells in A, when 100 nM DHT and 2 mM Ca^2+^ were applied and baseline for every cell was subtracted. E) PSA concentration in the media 10 min after stimulation with 100 nM DHT (double determination in n = 5 experiments) and PSA concentration in the media 24 h after stimulation with 100 nM DHT (double determination in n = 3 experiments) when cells were transfected with control RNA or Orai1 siRNA.

In order to investigate molecular components of DHT induced SOCE, we used the prostate cancer cell line LNCaP. In a siRNA based assay in LNCaP cells that were cultured in hormone deprived media for 48 h, down-regulation of SOCE components STIM1, STIM2, Orai1, Orai3 or Orai1 and Orai3 led to an overall decrease of DHT induced SOCE when compared to control RNA treated cells (solid lines, Figure 2A). Gene expression levels and efficiency of down-regulation from cells cultured in hormone deprived media for 48 h are shown in Supplementary Figure 2A and B. Down-regulation of STIM1, Orai1, Orai3 or Orai1 and Orai3 results in a significant decrease of Ca^2+^ influx rate (Figure 2B), Ca^2+^ peak (Figure 2C) and Ca^2+^ plateau (Figure 2D) of SOCE. Down-regulation of STIM2 significantly increased Ca^2+^ influx rate (Figure 2B), possibly due to a loss of STIM2 suppressing function of STIM1 as described earlier by [42]. Interestingly, STIM2 down-regulation also significantly decreased Ca^2+^ peak (Figure 2C) and Ca^2+^ plateau (Figure 2D) of SOCE. Global store-depletion by tg results in a higher Ca^2+^ signal upon Ca^2+^ release and higher SOCE when compared to DHT induced SOCE (black dotted line (tg) versus black solid line (DHT), Figure 2A). Down-regulation of Orai3 does not decrease but significantly increase Ca^2+^ influx rate, Ca^2+^ peak and Ca^2+^ plateau of tg-induced SOCE (Figure 2A and supplementary Figure 2C).

We next tested for Orai1's contribution to rapid androgen induced PSA release. Rapid DHT signaling increased basal PSA production up to 20% in LNCaP cells [8]. Comparison of PSA release from LNCaP cells transfected with control RNA (0.48±0.08 ng·mL^−1^·10^6^ cells^−1^) or with Orai1 specific siRNA (0.39±0.07 ng·mL^−1^·10^6^ cells^−1^) demonstrates that DHT induced PSA release depends on Orai1 whereas long-term gene expression (after 24 h) dependent PSA release appears to be independent on Orai1 (Figure 2E). In summary, knock-down molecular components of I_CRAC_ results in decreased Ca^2+^ signaling upon DHT stimulation. Down-regulation of main component of CRAC channel component Orai1 reduces DHT induced PSA release.

### ICRAC exhibits a unique 2-APB specific electrophysiological profile in hPEC

**Figure 3:**
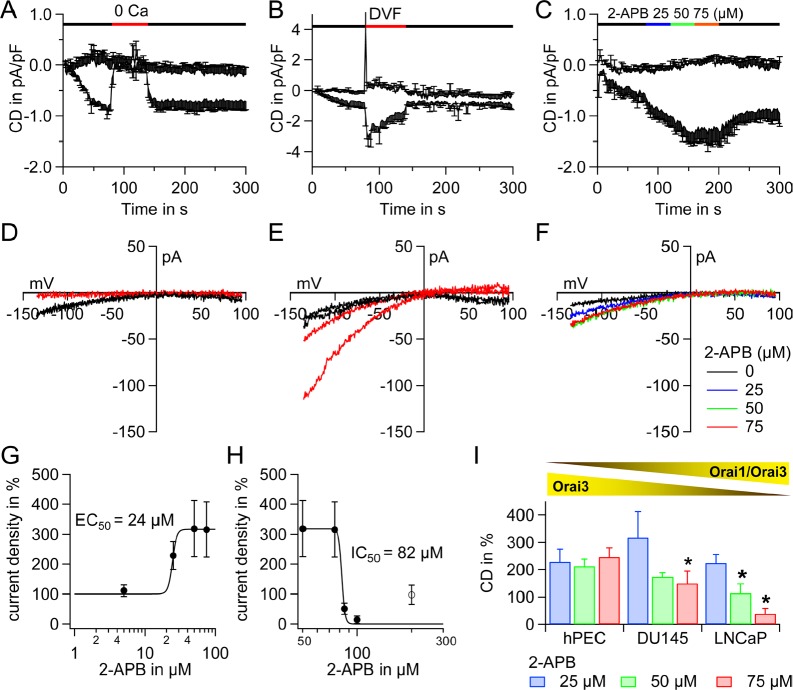
Electrophysiological and pharmacological characterization of ICRAC in hPEC and prostate cancer cell lines A Time course of I_CRAC_ evoked in hPEC by 50 μM IP_3_ and 10 mM BAPTA in the patch pipette and 20 mM Ca^2+^ Ringer in the bath. A 0 mM Ca^2+^ Ringer (0 Ca) was applied as indicated by the red bar (n = 4). B Same as in A, but a divalent free (DVF) external Ringer was applied as indicated by the red bar (n = 4). C Same as in A, but 2-APB was applied as indicated (n = 5). D corresponding IVs to A. E corresponding IVs to B. F corresponding IVs to C. G Current densities when 2-APB was applied were normalized to IP_3_ induced current at t = 80 s in %. 2-APB induced current potentiation was analysed with a dose-response fit function and an EC_50_ of 24 μM for potentiation was determined. H Same as G but 2-APB induced current inhibition was analysed. Data were fitted with a dose-response fit function and an IC_50_ of 82 μM for inhibition was determined. I Statistical analysis of 2-APB induced pharmacological profile of hPEC from cells in Figure 3C, DU145 (n=6) and LNCaP (n=6) from experiments performed as in Figure 3C.

Next, we tested for the properties of I_CRAC_ in hPEC in patch clamp experiments in order to investigate typical electrophysiological hallmarks of I_CRAC_. We evoked I_CRAC_ in a 20 mM Ca^2+^ Ringer solution by adding 10 mM BAPTA and 50 μM inositol-1, 4, 5-trisphosphate (IP_3)_ to the patch pipette. In these cells I_CRAC_ is ~ 0.5 – 1 pA/pF (Figure 3A, 3B and 3C). Application of 0 mM Ca^2+^ abolishes I_CRAC_ (Figure 3A, current-voltage curves IVs shown in Figure 3D) and upon application of divalent free solution (DVF) I_CRAC_ exhibits large inwardly rectified Na^+^ currents (Figure 3B, IVs shown in Figure 3E). These characteristics are in very good agreement to the literature about native CRAC channels (as discussed below). In many native cells as well as in STIM1/Orai1 overexpression systems, application of 2-APB (50 μM) amplifies and subsequently blocks I_CRAC_ (EC_50_ ~3 – 4 μM, IC_50_ ~ 8 – 10 μM, [43, 44]). Surprisingly, the 2-APB specific electrophysiological profile of hPEC (Figure 3C and Supplementary Figure 3A, IVs shown in Figure 3F and Supplementary Figure 3D and 2-APB induced dose-responses Figure 3G and H) differs from what is described for native CRAC channels and STIM1/Orai1 overexpression systems. The EC_50_ for potentiation is ~24 μM (Figure 3G) and the IC_50_ for inhibition is 82 μM (Figure 3H). The Orai1:Orai3 ratio in prostate cancer cell lines LNCaP und DU145 is elevated compared to hPEC (Orai1:Orai3 = 17±0.9, n = 4, in DU145 and Orai1:Orai3 = 26±0.9, n = 4, in LNCaP calculated from gene expression levels shown in Supplementary Figure 4A). Identical to the experiment in hPEC (Figure 3C and 3F) we determined the 2-APB induced electrophysiological profile in prostate cancer cell lines DU145 (Supplementary Figure 3B and 3E) and LNCaP (Supplementary Figure 3C and 3F). The statistical analysis in Figure 3I reveals a significant difference in pharmacological profile in DU145 upon application of 75 μM 2-APB and a significant difference in pharmacological profile in LNCaP upon application of 50 or 75 μM 2-APB when compared to hPEC from non-tumorous tissue.

As Orai3 is largely expressed in hPEC we next wanted to test if heteromeric Orai1/Orai3 channels might be responsible for the 2-APB specific electrophysiological profile.

### Orai3 is a regulator of SOCE and is responsible for the 2-APB specific electrophysiological profile of ICRAC in LNCaP cells

**Figure 4:**
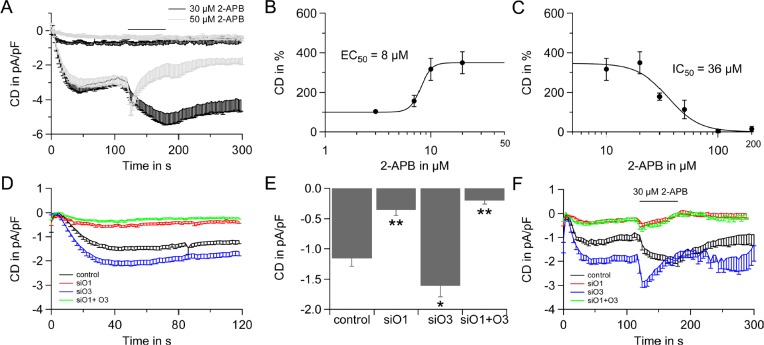
2-APB specific electrophysiological profile of LNCaP cells and the role of Orai3 A) Time course of I_CRAC_ evoked in LNCaP cells by 50 μM IP_3_ and 10 mM BAPTA in the patch pipette and 20 mM Ca^2+^ Ringer in the bath. 2-APB was applied as indicated (30 μM, n = 10, black line and 50 μM, n = 10, grey line). B) Dose response for 2-APB induced potentiation, EC_50_ = 8 μM. C) Dose response for inhibition of I_CRAC_ by 2-APB, IC_50_ =36 μM. D) Time course of I_CRAC_ evoked as in A from LNCap cells transfected with non-silencing control RNA (control, black, n = 21), transfected with Orai1 siRNA (O1, red, n = 30), Orai3 siRNA (O3, blue, n = 37) or Orai1 and Orai3 siRNA (siO1+O3, green, n = 29). Some of the cells in D are also shown in F. E) Current density at 120 s and statistical analysis for cells from D. F) Time-course of I_CRAC_ evoked as described in A when 30 μM 2-APB is applied on cells transfected with control RNA (control, black, n = 11), transfected with Orai1 siRNA (siO1, red, n=11), Orai3 siRNA (siO3, blue, n = 8) or Orai1 and Orai3 siRNA (siO1+O3, green, n = 9).

Given the extraordinary 2-APB specific electrophysiological profile of hPEC (Figure 3), the low Orai1:Orai3 ratio of ~4 (Figure 1) and Orai3's property to enhance Ca^2+^ currents upon 2-APB application [44-48] we tested the ability of Orai3 to shape the 2-APB specific electrophysiological profile of I_CRAC_ in the prostate cancer cell line LNCaP as LNCaP are less delicate to patch after transfection than hPEC. When 2-APB is applied in a concentration of 30 μM, I_CRAC_ is enhanced and application of 50 μM 2-APB results in current enhancement followed by an incomplete current block (Figure 4A, IVs are shown in Supplementary Figure 4C and 4D) as has previously been shown by [49]. In these cells, I_CRAC_ exhibits an EC_50_ of 8 μM (Figure 4B) and an IC_50_ of 36 μM (Figure 4C). Figure 4D shows I_CRAC_ in a siRNA based assay, when Orai1, Orai3, or both proteins are down-regulated. Analysis of IP_3_ induced currents show, that down-regulation of Orai3 significantly increases current size, while down-regulation of Orai1 or Orai1 and Orai3 significantly decreases the current size (Figure 4D and 4E). Upon siRNA based knock-down residual gene expression on mRNA level range from 11±5 % to 32±7 % (Supplementary Figure 4B).

Whereas I_CRAC_ in control RNA transfected cells is amplified upon application of 30 μM 2-APB, down-regulation of Orai1 or Orai1 and Orai3 results in an almost complete loss of 2-APB induced current. The fact that both knock-down conditions give the same result implies that Orai1 is the stringent requirement for a functional SOCE and that in LNCaP cells the CRAC channel likely exists as a heteromeric Orai1/Orai3 channel. Down-regulation of Orai3 introduces a 2-APB induced block of I_CRAC_ that is characteristic for STIM1/Orai1 mediated currents (Figure 4F). Thus, high expression levels of Orai3 shape a unique pharmacological profile for SOCE and Ca^2+^ signaling via specific CRAC channels in prostate cancer could be manipulated by substances selective for a specific channel composition.

### Relative gene expression of STIM1, STIM2, Orai1, Orai2 and Orai3 in non-tumorous and tumorous tissue from 13 patients with different Gleason score

**Figure 5:**
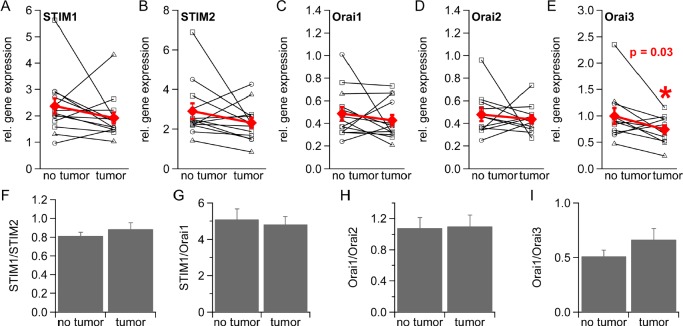
Gene expression of STIM1, STIM2, Orai1, Orai2 and Orai3 in healthy and tumorous human prostate tissue Relative gene expression of STIM1 (n = 13), STIM2 (n = 13), Orai1 (n = 13), Orai2 (n = 11) and Orai3 (n = 11) in healthy and tumorous tissue from prostate cancer patients normalized to the reference gene TBP and sorted by Gleason Score (○= 6, □= 7 and △= 8) (A-E) F) STIM1:STIM2 ratio. G) STIM1:Orai1 ratio. H) Orai1:Orai2 ratio. I) Orai1:Orai3 ratio.

We were interested in possible changes of I_CRAC_ component expression levels in prostate cancer as I_CRAC_ is reduced in prostate cancer resulting in several cancer hallmark functions. We thus determined relative STIM and Orai expression levels in non-tumorous and tumorous tissue from 13 prostate cancer patients by qRT-PCR, from which 13 expressed detectable levels of STIM1, STIM2 and Orai1 and 11 detectable levels of Orai2 and Orai3 (see methods). We find an over-all down-regulation of all I_CRAC_ components when gene expression is normalized to TBP (Figure 5A, 5B, 5C, 5D and 5E) or RNAPol (Supplementary Figure 5A, 5B, 5C, 5D and 5E). The different Gleason scores of prostate cancer tumors is indicated by symbols as described in the figure legend. For analysis we pooled data from patients with different Gleason scores. Orai3 is significantly down-regulated when gene expression levels are normalized to TBP (Figure 5E, p = 0.03 and p = 0.04 when gene expression levels are normalized to RNAPol, Supplementary Figure 5E). Levels of STIM1:STIM2, STIM1:Orai1, and Orai1:Orai3 gene expression ratios are slightly changed in tumorous tissue (Figure 5F, 5G and 5I and Supplementary Figure 5F, 5G and 5I) whereas the Orai1:Orai2 ratio remains unchanged (Figure 5H and Supplementary Figure 5H).

Our data suggest a down-regulation of I_CRAC_ components in prostate cancer and support the concept of low Ca^2+^ signaling in prostate cancer cells. Orai3 is significantly down-regulated and the decrease in Orai1:Orai3 ratio might reflect a different stoichiometry of Orai1/Orai3 subunits in CRAC channel that open the possibility for specific therapeutic targeting in prostate cancer.

## DISCUSSION

I_CRAC_ mediates several cellular functions such as cell cycle regulation, proliferation and apoptosis [26]. In prostate cancer, I_CRAC_ is well-known to be off-balance [50] and I_CRAC_ channels together with a variety of other Ca^2+^-transporting enzymes are under investigation as therapeutic targets [51, 52].

Our results uncover that STIM1 and Orai1 are I_CRAC_'s major molecular components in hPEC and STIM2, Orai2 and Orai3 are also expressed. I_CRAC_ channels are thought to exist either as tetramers [35-40] or as hexamers [41] and the low Orai1:Orai3 ratio of 4.3 supports the concept of heteromeric Orai1/Orai3 channels in hPEC.

I_CRAC_ in hPEC exhibits high Ca^2+^ selectivity and large monovalent currents in the absence of divalent ions comparable to native CRAC currents from Jurkat T cells, rat basophilic leukaemia cells (RBL) and mast cells [53-55]. The 2-APB specific electrophysiological profile in hPEC is unique with high EC_50_ and IC_50_ values (EC_50_ = 24 μM and IC_50_ = 82 μM) when compared to I_CRAC_ in a Jurkat T-cell line (EC_50_ = 3 μM and IC_50_ = 10 μM [43]) and STIM1 Orai1 overexpression systems (EC_50_ = 4 μM and IC_50_ = 8 μM [44]). Investigation of the 2-APB specific electrophysiological profile in a siRNA based assay in LNCaP cells suggest Orai1/Orai3 heteromeric channels as molecular basis for this unique pharmacology.

We find a significant down-regulation of Orai3 gene expression in tumorous tissue when compared to non-tumerous tissue from prostate cancer patients and an increased Orai1:Orai3 ratio. In addition, a comparison of Orai1:Orai3 gene expression ratios and electrophysiological profiles upon application of 2-APB in prostate cancer cell lines LNCaP, DU145 and hPEC support the idea of low levels of Orai3 in prostate cancer, although Orai3 is not reduced per se in cancer. In breast cancer tissue, Orai3 is up-regulated when compared to healthy tissue and its signaling includes cell cycle progression, apoptosis resistance, the mitogen-activated protein (MAP) kinase pathway and tumor formation [56-59]. The altered composition of CRAC channels in prostate cancer with a shift in Orai1:Orai3 ratio and distinct pharmacological profiles open the possibility to selectively manipulate I_CRAC_ activity in cancer cells (e.g. to higher Ca^2+^ signals and thereby drive cancer cells into apoptosis) without effecting Ca^2+^ signals in non-cancerous cells.

Targeting mAR and mAR induced signaling pathways is an intriguing strategy in the development of therapeutic approaches in prostate cancer [13]. So far, the androgen-induced increase of intracellular Ca^2+^ has been proposed to be mediated via Ca^2+^ store-depletion and L-type Ca^2+^ channels and to involve a pertussis sensitive G protein-coupled receptor [14, 60]. Evidence has accumulated that mAR activation leads to production of IP3 [61] and mAR induced IP_3_ production leads to the binding of IP_3_ to the IP_3_ receptor and the subsequent release of Ca^2+^ from intracellular Ca^2+^ stores [62, 63]. In 1992, Hoth and Penner demonstrated that Ca^2+^ store depletion by IP_3_ triggers I_CRAC_ [64]. Here, we demonstrate that rapid DHT signaling induces Ca^2+^ influx via CRAC channels in hPEC and a knock-down of the pore forming I_CRAC_ channel subunit Orai1 results in a dramatic reduction of mAR induced Ca^2+^ transients in hPEC. In addition, Ca^2+^ signaling via Orai1 functionally supports PSA release in rapid DHT response.

mAR exhibit higher expression levels in human prostate carcinoma cells when compared to non-tumorous and hyperplastic cells related to the Gleason score of the tumor [6, 7]. Higher expression levels of mAR are likely to increase store-depletion that is below maximum at DHT concentrations of 100 nM in LNCaP cells (compare store-depletion in Figure 2A, DHT vs tg) due to an elevated IP_3_ production. Patch clamp and imaging experiments indicate down-regulation of Orai3 results in elevated SOCE and I_CRAC_ when Ca^2+^ stores are heavily depleted by either tg or IP_3_ (Figure 2A, 4D and 4E and Supplementary Figure 2C).

We suggest that high mAR expression levels lead to stronger store depletion and in combination with Orai3 down-regulation to higher Ca^2+^ signals in prostate cancer. Once induced these elevated Ca^2+^ signals could bear the potential to counteract cancer hallmark functions that are characterized by low Ca^2+^ signaling e.g. uninhibited proliferation and inability to induce apoptosis. Thus, selective enhancement of I_CRAC_ channels in prostate cancer cells can be a promising approach in the development of mAR therapy.

At the moment a novel therapeutic approach against prostate cancer is tested in a clinical trial [65], tg coupled to a chemical cage that is specially cleaved of by prostate specific membrane antigen (PSMA) a prostate specific protease [66]. This so-called smart bomb is active only in prostate cancer cells. I_CRAC_ channels might be pharmacologic targets for the treatment of prostate cancer if they can be selectively manipulated without affecting I_CRAC_ channels in healthy cells. Thus, future therapies could include smart bombs on prostate cancer-specific I_CRAC_ channels.

## MATERIAL & METHODS

### Cell culture and prostate tissue collection

Prostate cancer lines Lymph Node Carcinoma of the Prostate (LNCaP) and DU145 were purchased from American Type Cell Culture Collection (ATCC, Rockville, MD, USA) and cultured with RPMI Medium 1640 (Life Technologies) supplemented with 10 % FCS and 1 % Pen/Strep (Life Technologies).

Prostate tissue was obtained from prostectomy specimens (Ethics approval 168/05, Ärztekammer des Saarlandes).

Human prostate epithelial cells (hPEC) were isolated with slight modifications according to [67]. Pieces from healthy tissues are washed in phosphate buffer solution and cut into cubes with a side length of 1 mm and placed into cell culture flasks. These small tissue cubes are wetted with PrEBM (Prostate Cell Basal Medium, #CC-3165, Lonza) supplemented with PrEGM Single Quots Supplements (#CC-4177, Lonza). Under these conditions prostate epithelial cells start to form a layer around the tissue piece after 2 to 6 days. After an adequate cell layer has been formed, pieces are removed and cells are taken in culture. For passaging Cell Dissociation Solution (#C5789, Sigma) was used to detach cells and cells were not grown in media described above.

For comparison of expression levels of target genes via qRT-PCR in healthy and cancer prostate tissues primary prostate adenocarcinoma samples, which were obtained after radical prostatectomy from thus far untreated prostate cancer patients, were investigated. Following prostatectomy, the specimens were dissected by a pathologist, snap frozen, and stored at −80°C. Only samples containing >50% tumor cells were included in the study. In the present subset of 13 prostate cancer samples were classified with Gleason score as indicated.

### Quantitative RealTime-PCR (qRT-PCR)

Total RNA from LNCaP, DU145 and hPEC was isolated with TRIzol Reagent (Life Technologies) and from prostate cancer tissue with RNeasy Mini kit (Qiagen). For reverse transcription 0.8 μg of isolated total RNA was used.

0.5 μl complementary DNA (cDNA) and 300 nM primer were used in a QuantiTect SYBRgreen kit (Qiagen). PCR conditions were as follows: 15 min at 95°C; 45 cycles, 30 s at 95°C; 45 s at 58°C; and 30 s at 72°C and finally a cycle (60 s, 95°C; 30 s 55°C; 30 s 95°C) to determine specificity by a dissociation curve using the MX3000 cycler (Stratagene). Expression of target genes were normalized to the expression of the reference genes RNA polymerase II (RNAPol, NM_000937) and/or TATA box binding protein (TBP, NM_003194). Primer sequences were as follows for Orai1 5'atgagcctcaacgagcact3' (forward) and 5'gtgggtagtcgtggtcag3' (reverse), for Orai3 were 5'gtaccgggagttcgtgca3' (forward) and 5'ggtactcgtggtcactct3' (reverse), for STIM1 were 5' cagagtctgcatgaccttca 3' (forward) and 5' gcttcctgcttagcaaggtt 3' (reverse), for STIM2 were 5' gtctccattccaccctatcc 3' (forward) and 5' ggctaatgatccaggaggtt 3' (reverse), TBP were 5' cggagagttctgggattgt 3' (forward) and 5' ggttcgtggctctcttatc 3' (reverse) and RNAPol were 5' ggagattgagtccaagttca 3' (forward) and 5' gcagacacaccagcatagt 3' (reverse).

### Ca2+ Imaging

Bath solution contained (in mM): 155 NaCl, 4.5 KCl, 2 MgCl_2_, 10 glucose, 5 HEPES (pH 7.4 with NaOH), and CaCl_2_ was adjusted as indicated. Stock solutions of tg were prepared in DMSO at a concentration of 1 mM and of DHT in ethanol at a concentration of 5 mM. For Ca^2+^ imaging assays (see below) LNCaP cells and hPEC were cultured for 48 h in hormone deprived RPMI media (Sigma R7509), supplemented with 10% charcoal stripped FBS (Sigma F6765) and 2 mM L-glutamine, when DHT was used as a stimulus.

Cells were plated on glass cover slips for at least 24 h and loaded with 1 μM Fura-2/AM at 37°C for 20 min. Afterwards glass coverslips were placed in a perfusion chamber in a Zeiss Axio Observer.A1 fluorescence microscope equipped with a “Plan-Neofluar” 20x/0.4 objective (Zeiss). The excitation light generated by a Polychrome V in a TILL Photonics realtime imaging system alternated at 340 and 380 nm and the exposure time was set to 50 ms in each channel. Light intensity at emission wavelength 440 nm was detected every 5 s and digitized by a charge-coupled device camera (Q-Imaging Retiga 2000RV). Data was analysed with TILL Vision software. Intracellular Ca^2+^ concentration was calculated from the equation [Ca^2+^]_i_ = K(R − R_min_)/(R_max_ – R) in which K, R, R_min_ and R_max_ where determined in the corresponding in situ calibration for hPEC and LNCaP cells according to [68].

### Electrophysiology

Tight seal whole-cell patch clamp experiments were performed with a Patchmaster software controlled EPC-10 patch-clamp amplifier (HEKA). The fire-polished patch-pipettes had resistances between 2 and 4 ΩM. Voltage ramps of 50 ms duration were delivered every 2 s from a holding potential of 0 mV spanning −150 mV to +100 mV for hPEC and −150 mV to +150 mV for the LNCaP cells. Capacitive currents were determined and corrected before each voltage ramp. Current sample rate was 3 kHz and data were filtered at 1 kHz. All voltages were corrected for a liquid junction potential of −10 mV. For analysis leak currents before current activation were subtracted and currents extracted at −130 mV and 80 mV and blotted vs time.

The bath solutions contained in mM: 95 NaCl, 2.8 KCl, 20 CaCl_2_, 2 MgCl_2_, 10 HEPES, 10 TEACl, 10 CsCl, 10 glucose for LNCaP cells and 120 NaCl, 10 TEACl, 20 CaCl_2_, 2 MgCl_2_, 10 HEPES, 10 glucose for hPEC. The pH was adjusted with NaOH to 7.2 and osmolarity was 300 mosmol/L for cell lines and 330 for primary prostate epithelial cells. In 0 mM Ca^2+^ solution CaCl_2_ was omitted and in divalent free solution (DVF) MgCl_2_ and CaCl_2_ were replaced by 10 mM EDTA, osmolarity was adjusted to 330 mosmol/L with glucose. In 2-APB experiments 2-APB was added as indicated. Pipette solution contained in mM: 120 Cs glutamate, 10 BAPTA, 10 HEPES, 3 MgCl_2_ and 0.05 IP_3_ for LNCaP cells and 140 Cs glutamate, 8 NaCl, 10 BAPTA, 10 HEPES, 3 MgCl_2_ and 0.05 IP_3_ for hPEC. For reasons of comparability DU145 and LNCaP in Fig. 3 and S3 were patched under the same conditions as hPEC.

### Data analysis and statistics

Data were analyzed with TILLVision (TILL Photonics), Fitmaster 2.35 (HEKA), Igor Pro (Wavemetrics), and Microsoft Excel (Microsoft). Data are given as means ± SEM. Asterisks indicate significance determined by an unpaired, two-sided Student's t-test *p < 0.05, **p < 0.01, ***p < 0.001. Significance of changes in PSA release assays was tested with a one-sided unpaired t-test. Significance of changes of relative gene expression in tissue probes from patients was analyzed with a paired t-test. EC_50_ and IC_50_ values were determined by a fit with Hill's equations (least-squares method). For qRT-PCR relative expression was calculated according to the ΔCq method (2^−ΔCq^) where Cq values are determined with the MX3000 software and excluded from analysis when they exceeded 35 cycles.

### Small interfering RNA transfection (siRNA)

SiRNA tranfections were perfomed with 0.12 nmol of siRNA with a Nucleofector II (Lonza) nucleofector using Nucleofector transfection Kit R (Lonza) according to manufacturer' instructions. All siRNAs were from Qiagen or Microsynth and were in part modified according to [69]. Orai1 siRNAs were Hs_TMEM142A_1, #SI03196207 [sense: 5'OMeC-OMeG-GCCUGAUCUUUAU-CG-d-(UCU)-OMeU-OMeT-OMeT3'; antisense: 3'OMeG-OMeC-CGGACUAGAAA-UA-GCAGAd(A)5'] and Hs_TMEM142A_2, #SI04215316 [sense: 5'OMeC-OMeA-ACAUCGAGGCG-GUG-A)d(GCA)OMeA-OMeT-OMeT3'; antisense: 3'OMeG-OMeT-UGUAGCUCCGCCA-CUCGUd(U)5']. Orai3 siRNAs were Hs_TMEM142C_2, # SI04174191 [sense: 5′OMeC-OMe-A-CCAGUGGCUACCUCCd(CUU)OMeA-OMeTOMeT3′; antisense: 3′OMeG-OMeT--GG-UCACCGAUGGAGGGAAd(U)5′] and Hs_TMEM142C_5, #SI04348876 [sense: 5′OMeT-OMeC-CUU-AGCCCUUGAAAU)d(ACA)OMeA-OMeT-OMeT3′; antisense: 3′OMeA--OMeG-GA-A-U-CGGGAACUUUAUGUd(U)5′]. STIM1 siRNAs were Hs_STIM1_5, # SI03235442 [sense: 5'OMeU-OMeGAGG-UGGAGGUGCAAUd(AUU)dOMeA-dOMeT-dOMeT3′; antisense: 3'OMeA-OMeC-U-C-CACCUCCACGUUAUAAd(U)5'] and Hs_STIM1_6, # SI04165175 [sense: 5'OMeC-OMeU-GGUGG-UGUCU-A-UCGUd(UAU) OMeU-OMeT-OMeT3'; antisense: 3'OMeG-OMeA-CCACCA-CA-G-AUAGCAAUAd(A)5'].

STIM2 siRNAs were Stim2_6 (Microsynth), [sense: 5'UAAGCAGCAUCCCACAU-GAdT-d-T-3'; antisense: 3'dTdTAUUCGUCGUAGGGUGUACU5'] and Stim2_7 (Microsynth), [sense-: 5'AAUUUAGAG-CGCAAAAU-GAdTdT3'; antisense: 3'dTdTUUAA-AUCU-CGCGUUUUACU5'] and Stim2_8 (Microsynth) [sense: 5'GUGCACGAACCUUCAU-U-U-A-d-Td-T3'; antisense: 3'dTdTCACGUGCUUGGAAGUAAAU5']. Non-silencing RNA were MS_control_mod [sense: 5'OmeA-OMeA-AGGUAGUGUAAUCGCd(CUU)OMeG-OmeT-OMeT3'; antisense: 3'OmeT-OmeT-UCCAUCACAUUAGCGGAAdC 5'].

### Determination of prostate specific antigen (PSA)

LNCaP cells were transfected with either control or Orai1 specific siRNA and seeded in 6-well plates. After 24 h medium was replaced by hormone deprived medium for 48 h. After 100 nM DHT has been added, 250 μl of supernatant was removed at the time points indicated and total PSA within the supernatant was determined in an ECLIA (ElectroChemiLuminescence ImmunoAssay) using a cobas system (Roche). PSA was determined in ng/mL and normalized to 10^6^ cells.

## Supplementary Figures


