# Supplementary material for: ICRAC controls the rapid androgen response in human primary prostate epithelial cells and is altered in prostate cancer
Source: Oncotarget. 2013 Oct 28;4(11):2096–107. doi: 10.18632/oncotarget.1483 (PMC3875772; doi:10.18632/oncotarget.1483)
Supplement: Supplementary file 1 [file oncotarget-04-2096-s001.pdf]

ICRAC controls the rapid androgen response in human primary prostate epithelial cells and is altered in prostate cancer - Holzmann et al

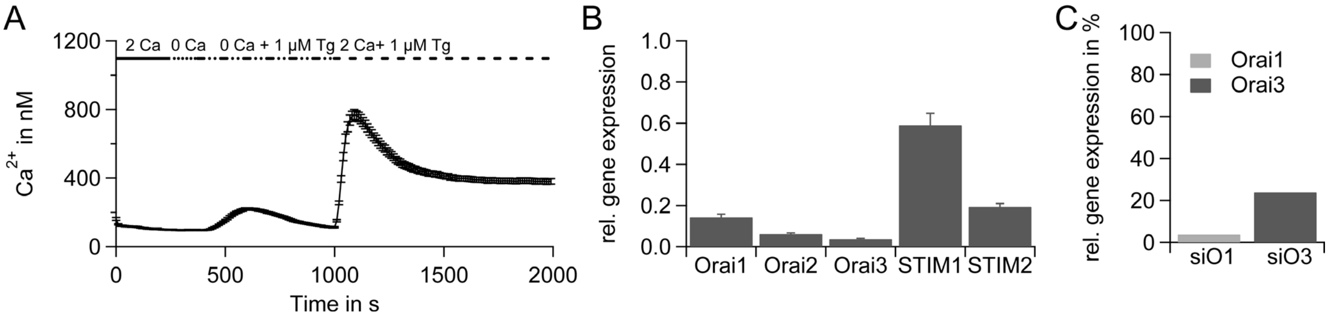

Supplementary Figure 1: A Average intracellular Ca^{2+} responses (±SEM) from a Fura-2 based Ca^{2+} imaging assay before and after store depletion by addition of 1 µM tg, n = 111 in hPEC. Ca^{2+} concentration is indicated in mM. B qRT-PCR analyses of Orai1, Orai2, Orai3, STIM1 and STIM2 expression levels from hPEC from 17 different patients normalized to RNA polymerase II (RNAPol) expression as reference gene. C Efficiency of gene down-regulation in siRNA based assay in % in hPEC. Ratio between gene expression levels of Orai1 and Orai3 in siRNA transfected cells and in cells transfected with control RNA in %.

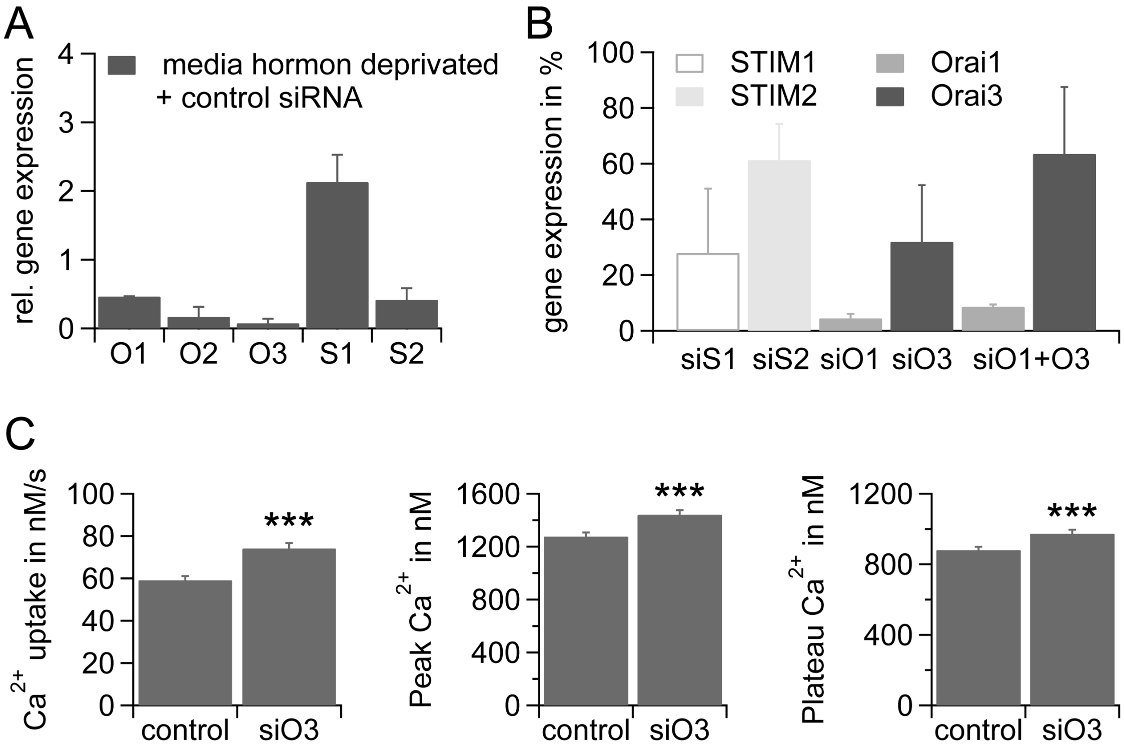

Supplementary Figure 2: A qRT-PCR analyses of Orai1, Orai2, Orai3, STIM1 and STIM2 expression levels from LNCaP cells that were hormone deprived for 48 h and transfected with control RNA (n = 3) normalized to TBP expression as reference gene. B Efficiency of gene down-regulation in siRNA based assay in % in cells in A. Ratio between gene expression levels of STIM1, STIM2, Orai1 and Orai3 in siRNA transfected cells (siS1, n = 2; siS2, n = 2; siO1, n = 2; siO3, n = 2 or siO1+O3, n = 2) and in cells transfected with control RNA in %. C Analysis of average Ca^{2+} influx rates, average Ca^{2+} peaks and average Ca^{2+} plateaus from cells in Fig. 2a, when stores were depleted with 1 µM tg.

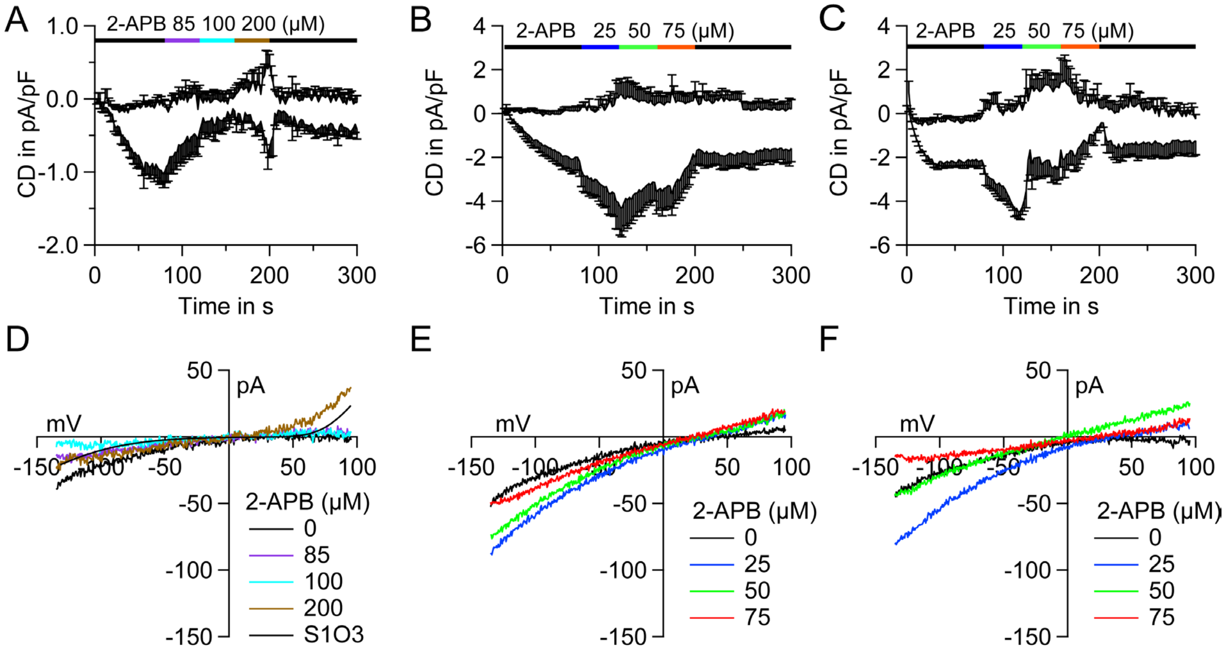

Supplementary Fig. 3: A Same as in Fig. 3c for DU145 cells (n = 6) B Same as in Fig. 3c for LNCaP cells (n = 6). C Same as in Fig. 3c for hPEC when 85 µM, 100 µM and 200 µM 2-APB were applied (n = 5) D IVs to A E IVs to B F IVs to C.

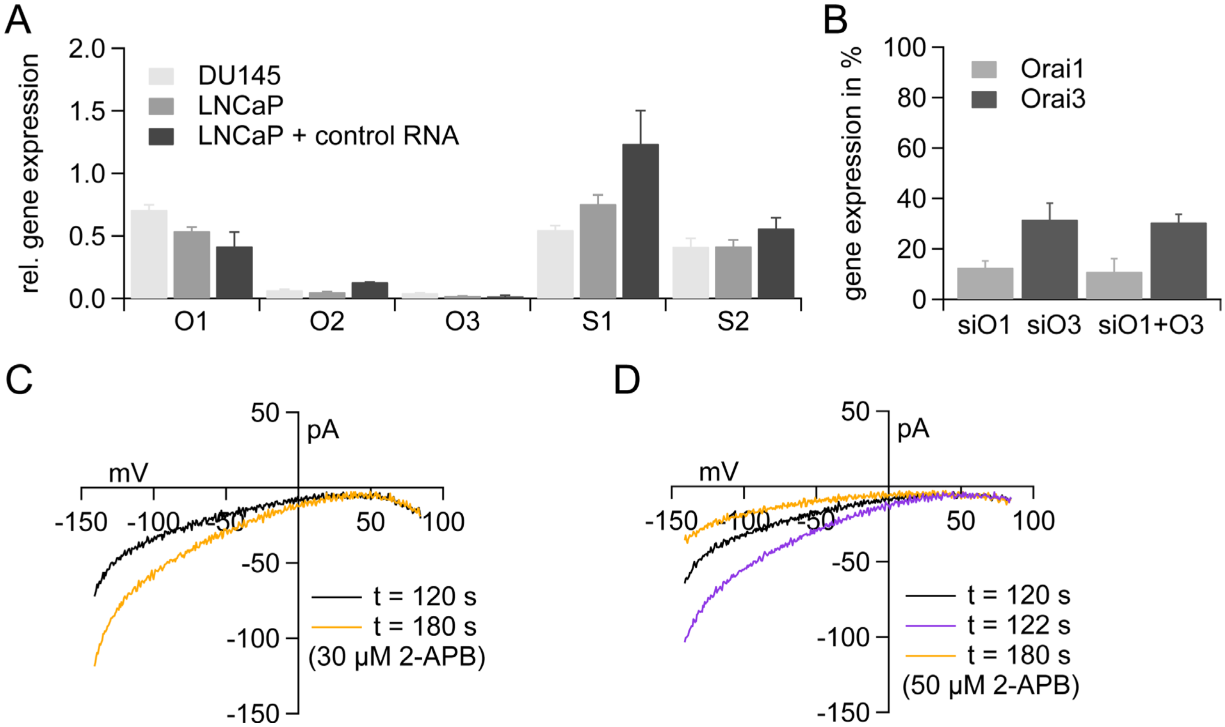

Supplementary Fig. 4: A qRT-PCR analyses of Orai1, Orai2, Orai3, STIM1 and STIM2 expression levels from non-transfected DU145 cells (light grey, n = 4), LNCaP cells (grey, n = 4) and LNCaP cells transfected with control RNA (dark grey, n = 3) normalized to TBP expression as reference gene. B Efficiency of gene down-regulation in siRNA based assey in %. Ratio between gene expression levels of Orai1 and Orai3 in siRNA transfected cells (siO1, n = 3; siO3, n = 3 or siO1+O3, n = 3) and in cells transfected with control RNA in %. C IVs corresponding to Fig. 4a when 30 µM 2-APB were applied. D IVs corresponding to Fig. 4a when 50 µM 2-APB were applied.

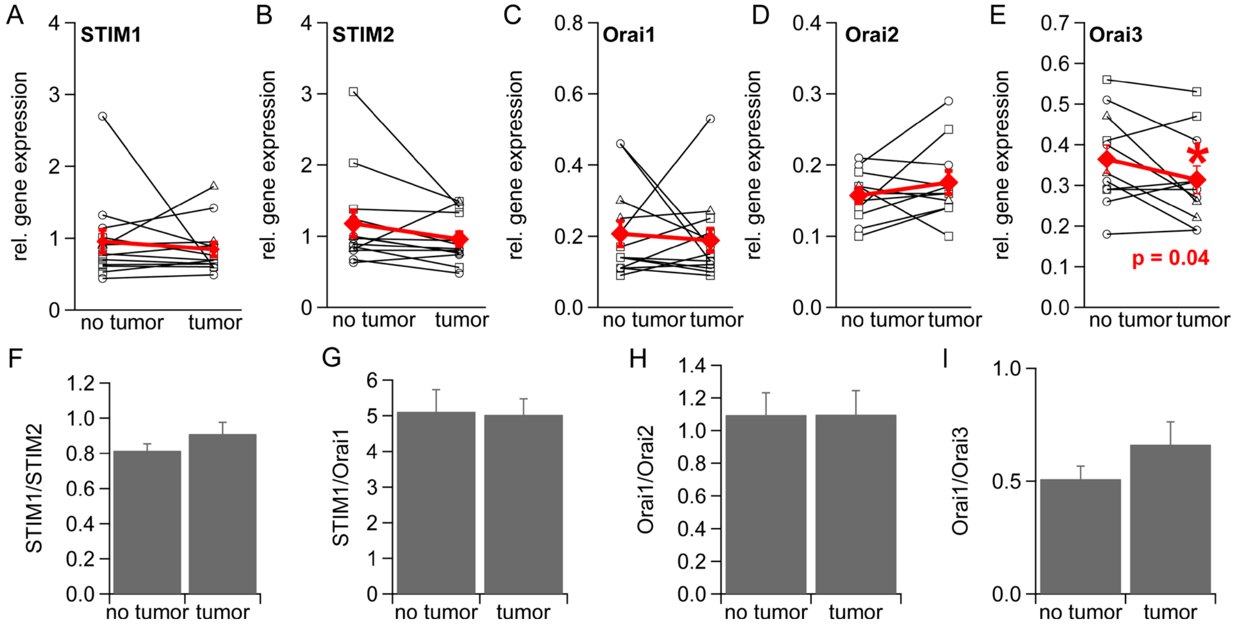

Supplementary Figure 5: Relative gene expression of STIM1 (n = 13), STIM2 (n = 13), Orai1 (n = 13), Orai2 (n = 11) and Orai3 (n = 11) in healthy and tumorous tissue from prostate cancer patients normalized to the reference gene RNAPol and sorted by Gleason Score (○ = 6, □ = 7 and △ = 8) (A-E) F STIM1:STIM2 ratio. G STIM1:Orai1 ratio. H Orai1:Orai2 ratio. I Orai1:Orai3 ratio.
